# *Bartonella* DNA in Dog Saliva

**DOI:** 10.3201/eid1312.070653

**Published:** 2007-12

**Authors:** Ashlee W. Duncan, Ricardo G. Maggi, Edward B. Breitschwerdt

**Affiliations:** * North Carolina State University College of Veterinary Medicine, Raleigh, North Carolina, USA

**Keywords:** Bartonella DNA, dog saliva, emerging pathogens, dispatch

## Abstract

*Bartonella* species, transmitted by arthropods or animal bites and scratches, are emerging pathogens in human and veterinary medicine. PCR and DNA sequencing were used to test oral swabs collected from dogs. Results indicated the presence of 4 *Bartonella* species: *B. bovis, B. henselae, B. quintana*, and *B. vinsonii* subspecies *berkhoffii*.

*Bartonella* species are being recognized as increasingly important bacterial pathogens in veterinary and human medicine. These organisms can be transmitted by an arthropod vector or alternatively by animal scratches or bites (*1*). Among the 11 species or subspecies known or suspected to be pathogenic in humans, 8 have been detected in or isolated from pet dogs or cats, thereby highlighting the zoonotic potential of these bacteria (*2*). In general, cats are implicated in the transmission of *Bartonella henselae*, typically resulting in cat-scratch disease; however, there have also been sporadic reports of *Bartonella* transmission by dogs (*3*–*5*). When *B. henselae* prevalence was evaluated in a population of 52 dogs, 4 dogs were seroreactive at reciprocal titers of 64 or 128, and *Bartonella*-positive PCR results were found in 3 of 52 blood samples, 5 of 9 oral swabs, and 5 of 9 nail clippings (*5*). Based on these reports and the recent recognition of *B. henselae* and *B. vinsonii* subspecies *berkhoffii* bacteremia in veterinarians and veterinary technicians who experience frequent cat and dog scratches and bites (*6*), we speculated that *Bartonella* species may be present in the saliva of dogs. The purpose of this study was to determine whether *Bartonella* DNA could be detected in oral swabs collected from dogs.

## The Study

As part of an ongoing study from November 2004 to December 2006 to investigate the prevalence of *Anaplasma, Bartonella*, and *Ehrlichia* infections in healthy golden retrievers and golden retrievers with lymphoma, a buccal swab was collected using a sterile cotton applicator. The swab was placed against the inside surface of the dog’s cheek. Saliva and tissue were collected by rolling the swab firmly against the cheek. Subsequently, the swab was placed into a sterile, no additive, Vacutainer (Becton Dickinson**,** Franklin Lakes, NJ, USA) serum tube and allowed to air dry for 10 to 15 minutes at room temperature before the tube was recapped.

Cells on the air-dried swab were resuspended in 500 μL of QuickExtract DNA Extraction Solution (EPICENTRE Biotechnologies, Madison, WI, USA), according to the manufacturer’s instructions. Total DNA was isolated using 200 μL of the QuickExtract resuspension, which was extracted through a QIAamp DNA Blood Mini-Kit (QIAGEN, Inc., Valencia, CA, USA) according to the manufacturer’s instructions. Similarly, total DNA was extracted from 200 μL of EDTA-anticoagulated whole blood using the QIAamp DNA Blood Mini-Kit.

Oral swabs and blood samples (n = 44 each) were screened for the presence of *Bartonella* by 2 previously described PCR methods (*7*). The first PCR targeted a fragment of the 16S-23S intergenic transcribed spacer (ITS) region; samples that were PCR positive for *Bartonella* DNA by the ITS primers were subsequently analyzed by a second PCR targeting the heme-binding protein gene, Pap31. Positive and negative controls were used in all processing steps, including DNA extraction. PCR amplicons were sequenced to identify species (Davis Sequencing, Davis, CA, USA). Sequence analysis and alignment with GenBank sequences were performed (AlignX, Vector NTI Suite 6.0, InforMax, Inc., Frederick, MD, USA). Additionally, serum samples were analyzed for IgG antibodies to *B. henselae* and *B. vinsonii* (*berkhoffii*) using an indirect immunofluorescence assay (IFA), as described previously (*8*). Reciprocal titers >64 were considered seroreactive.

Of the 44 dogs surveyed, oral swabs collected from 5 (11.4%) dogs were PCR-positive for *Bartonella* DNA. Sequencing indicated that 5 different *Bartonella* species or subtypes were present: *B. bovis*, *B. henselae*, *B. quintana*, and *B. vinsonii* subsp. *berkhoffii* types I and II (Table). PCR amplification and sequencing of blood samples from these 5 dogs showed *B. henselae* and *B. vinsonii* (*berkhoffii*) DNA in 2 dogs (Table). None of these 5 dogs was seroreactive to *B. henselae* or *B. vinsonii* (*berkhoffii*) antigens. Contamination was not detected in any of the negative control samples at any stage of processing or at any time during the study. As this work was part of an ongoing study of golden retrievers with and without lymphoma, dogs 1 and 2 had lymphoma; the remaining 3 dogs were clinically healthy (Table).

**Table:** PCR, DNA sequencing, and serologic results for the 5 dogs positive for *Bartonella* DNA in oral swabs*

Dog no.	*B. henselae* IFA	*B. vinsonii* (*berkhoffii*) IFA	PCR target	DNA sequence in blood	DNA sequence in oral swab
1	<16	<16	ITS, Pap31	*B. henselae* and *B. vinsonii* (*berkhoffii*) type II, *B. henselae*	*B. bovis,* negative
2	<16	<16	ITS, Pap31	*B. henselae, B. henselae*	*B. vinsonii* (*berkhoffii*) types I and II, *B. henselae* and *B. vinsonii* (*berkhoffii*) type II
3	32	<16	ITS, Pap31	Negative, negative	*B. vinsonii* (*berkhoffii*) type II, negative
4	<16	<16	ITS, Pap31	Negative, negative	*B. vinsonii* (*berkhoffii*) type II, negative
5	<16	<16	ITS, Pap31	Negative, negative	*B. quintana,* negative

*ITS, 16S–23S intergenic transcribed spacer region; Pap31, heme-binding protein gene; IFA, indirect immunofluorescence assay.

## Conclusions

These results demonstrate the presence of *Bartonella* DNA in oral swabs obtained from dogs. Notably, 3 *Bartonella* species and 2 *B. vinsonii* (*berkhoffii*) types were found in dog saliva. *B. bovis*, formerly referred to as *B. weissii*, was initially isolated from the blood of cats (*9*). Subsequently, this organism was isolated from the blood of cows in the United States, Europe, and Africa (*10*–*12*). To our knowledge, this is only the second known report of the detection of *B. bovis* DNA in a sample obtained from a dog (*13*). All 5 dogs in this study lacked serologic evidence of *Bartonella* infection, a finding which has been previously reported in bacteremic dogs and humans (*6**,**13**,**14*).

Previous studies have shown that targeting multiple *Bartonella* genes provides molecular evidence of coinfection with more than 1 *Bartonella* species or strain type (*6*,*7*,*13*). In the current work, the inability to confirm the ITS PCR results with a second PCR target has been previously reported by our laboratory (*6*,*13*,*14*) and likely reflects differences in PCR sensitivity, interference or inhibition of the PCR reaction by oral bacteria that are present in greater numbers than the *Bartonella*, or the lack of a known heme-binding protein gene in various *Bartonella* species, such as *B. bovis*. The limit of detection (LOD) of *Bartonella* ITS PCR is 2 copies/reaction, while the LOD of Pap31 assay is 10 copies/reaction. Further, although *B. henselae* has a detectable Pap31 protein (Table), several researchers in our laboratory have successfully isolated *B. henselae* strains that lack a PCR-detectable heme-binding protein (unpub. data). Upon recognition of the discordance between ITS and Pap31, additional genes such as 16S, *glt*A, and *rpo*B were targeted; however, these analyses were negative for *Bartonella* and resulted in nonspecific bacterial amplification. Because inhibition of ITS PCR was suspected due the presence of other oral bacteria, *Bartonella*-negative DNA extracts from oral swabs were spiked with *B. henselae* DNA at 1.5, 2.5, 5, and 10 (0.002 pg/μL) copies/reaction. Inhibition was detected at up to 5 copies/reaction, while the 10 copies/reaction sample was consistently amplified by the ITS primers.

These data, in conjunction with previous case reports (*3*–*5*), suggest that potentially viable *Bartonella* organisms may be transmitted to humans after a dog bite. The detection of DNA by PCR does not necessarily indicate the viability of *Bartonella* organisms. However, due to the extremely slow growth characteristics of *Bartonella* spp., isolation from the oral cavity does not seem feasible, because of competition with numerous other rapidly growing oral bacterial species. Recently, *Bartonella* DNA has been amplified from peripheral lymph nodes of healthy dogs (*14*). *B. henselae* was also amplified from salivary gland tissues from a dog with saladenitis (*15*). There are several plausible routes by which a *Bartonella* sp. could gain entry to the oral cavity. Future studies should determine if the tonsilar lymphoid tissues, salivary glands, or periodontal, gingival, or other oral tissues can serve as sources of *Bartonella* spp. contamination of canine saliva. As *Bartonella* infection may represent an occupational risk for veterinary professionals and others with extensive animal contact (*6*), additional studies should address the risk of transmission from dogs to humans following bite wounds.
